# A Topological Criterion for Filtering Information in Complex Brain Networks

**DOI:** 10.1371/journal.pcbi.1005305

**Published:** 2017-01-11

**Authors:** Fabrizio De Vico Fallani, Vito Latora, Mario Chavez

**Affiliations:** 1 Inria Paris, Aramis project-team, Paris, France; 2 CNRS UMR-7225, Sorbonne Universités, UPMC Univ Paris 06, Inserm, Institut du cerveau et de la moelle (ICM) - Hôpital Pitié-Salpêtrière, Paris, France; 3 School of Mathematical Sciences, Queen Mary University of London, London, United Kingdom; 4 Dipartimento di Fisica e Astronomia, Università di Catania and INFN, Catania, Italy; Ghent University, BELGIUM

## Abstract

In many biological systems, the network of interactions between the elements can only be inferred from experimental measurements. In neuroscience, non-invasive imaging tools are extensively used to derive either structural or functional brain networks *in-vivo*. As a result of the inference process, we obtain a matrix of values corresponding to a fully connected and weighted network. To turn this into a useful sparse network, thresholding is typically adopted to cancel a percentage of the weakest connections. The structural properties of the resulting network depend on how much of the inferred connectivity is eventually retained. However, how to objectively fix this threshold is still an open issue. We introduce a criterion, the efficiency cost optimization (ECO), to select a threshold based on the optimization of the trade-off between the efficiency of a network and its wiring cost. We prove analytically and we confirm through numerical simulations that the connection density maximizing this trade-off emphasizes the intrinsic properties of a given network, while preserving its sparsity. Moreover, this density threshold can be determined *a-priori*, since the number of connections to filter only depends on the network size according to a power-law. We validate this result on several brain networks, from micro- to macro-scales, obtained with different imaging modalities. Finally, we test the potential of ECO in discriminating brain states with respect to alternative filtering methods. ECO advances our ability to analyze and compare biological networks, inferred from experimental data, in a fast and principled way.

## Author Summary

Complex brain networks are mainly estimated from empirical measurements. As a result, we obtain networks where everything is connected to everything else through different strengths of interaction. Filtering procedures are typically adopted to prune weakest connections. However, network properties strongly depend on the number of remaining links and how to objectively fix such threshold is still an open issue. Here, we propose a criterion (ECO) to filter connectivity based on the optimization of fundamental properties of complex systems, i.e., efficiency and economy. Using ECO, investigators can analyze and compare connectomes in a fast and principled way, capturing network properties of different brain states to eventually quantify (re)organizational mechanisms underlying cognition and disease. Given its generality, we anticipate that ECO can also facilitate the study of networks in other fields, such as system biology.

This is a *PLOS Computational Biology* Methods paper.

## Introduction

Network science has provided a breakthrough in the analysis and modeling of biological systems with the aim to unlock molecular mechanisms behind human disease [1–3] and quantify brain (re)organization underlying behavior, cognition and mental disorders [4–6].

In part, this has been made possible by the increasing availability of tools that indirectly infer the structure of those networks from empirical measurements, thus bypassing the current lack of accurate and complete interaction maps [3, 7]. In system biology, functional links are estimated from transcriptional or phenotypic profiling, and genetic interactions by using measures such as Pearson correlation [8] or Granger causality [9].

In neuroscience, imaging tools such as magnetic resonance imaging (MRI) and electro/magnetoencephalography (E/MEG), are extensively used to map connections and/or interactions between different brain sites, i.e., the connectome [7, 10]. Brain connectivity methods are typically used to estimate the links between the nodes. While structural connectivity (SC) measures the probability to find axonal pathways between brain areas, typically from diffusion MRI, functional connectivity (FC) rather calculates the temporal dependence between remote neural processes as recorded, for instance, by functional MRI, EEG or MEG [4, 7].

At this stage, the resulting networks correspond to maximally dense graphs whose weighted links code for the strength of the connections between different nodes. Common courses in brain network analysis use thresholding procedures to filter information in these raw networks by retaining and binarizing a certain percentage of the strongest links (S1 Fig). Despite the consequent information loss, these procedures are often adopted to mitigate the incertainty of the weakest links, reduce the false positives, and facilitate the interpretation of the inferred network topology [3, 11].

At present, there’s no objective way to fix the value of such threshold. Because network properties significantly depend on the number of remaining links, scientists are obliged to explore brain network properties across a wide range of different candidate thresholds and eventually select one representative *a-posteriori* [12]. Concurrently, alternative approaches can be adopted to cancel spurious links emerging from third-party interactions [13–15], or statistically validate the estimated connections [7, 16, 17]. However, these procedures lack of precise rationale, are subject to arbitrariness (e.g., the choice of the statistical significance) and make difficult the comparison of network properties between many individuals or samples [11, 18]. Furthermore, these become extremely time-consuming when considering several large connectomes due to the computational complexity of graph quantities based on paths between nodes or on communities detection [19].

To circumvent these issues, we propose a topological criterion for selecting a threshold which captures the essential structure of a network while preserving its sparsity. Based on the optimal trade-off between two desirable but incompatible features—namely high global and local integration between nodes, and low connection density—this method is inherently motivated by the principle of efficiency and economy observed in many complex systems [20], including the brain [21].

## Results

### Filtering information as a network optimization problem

Global- and local-efficiency have revealed to be important graph quantities to characterize the structure of complex systems in terms of integration and segregation of information [22, 23].

Both structural and functional brain networks tend to exhibit relatively high values of global- and local-efficiency. At the same time they also tend to minimize, for economical reasons, the number of their links leading to sparse networks [21].

Thus, we propose to determine a density threshold that filters out the weakest links and maximizes the ratio between the overall efficiency of a network and its wiring cost. Notice that the definition of cost can have different connotations, e.g., the spatial distance between connected nodes [21]. Here, the cost in terms of number of links is a more general definition which also applies to non-spatially embedded networks (e.g., molecular interaction networks). We formally introduce a criterion to filter information in a given network by finding the connection density *ρ* that maximizes the quality function:

$$J=\frac{E_{g}+E_{l}}{\rho }$$ (1)

where *E*_*g*_ and *E*_*l*_ represent respectively the global- and local-efficiency of a network. By definition, the three quantities *E*_*g*_, *E*_*l*_ and *ρ* are normalized in the range [0, 1], and both *E*_*g*_ and *E*_*l*_ are non-decreasing functions of *ρ*. More details about the formulation of *J* can be found in the Material and Methods.

For both regular lattices and random networks, we proved analytically that the optimal density that maximizes *J* follows a power-law *ρ* = *c*/(*n* − 1), where *c* is a constant and *n* is the network size, i.e., the number of nodes in the network. More specifically, *c* = 3.414 for lattices and *c* = *e* = 2.718 for random networks, so that we have approximately *ρ* ≃ 3/(*n* − 1). Hence, to maximize *J*, these networks have to be sparse with an average node degree *k* ≃ 3 or, equivalently, with a total number of links *m* that scales as $m≃\frac{3}{2}n$ (S1 Appendix).

**Fig 1:**
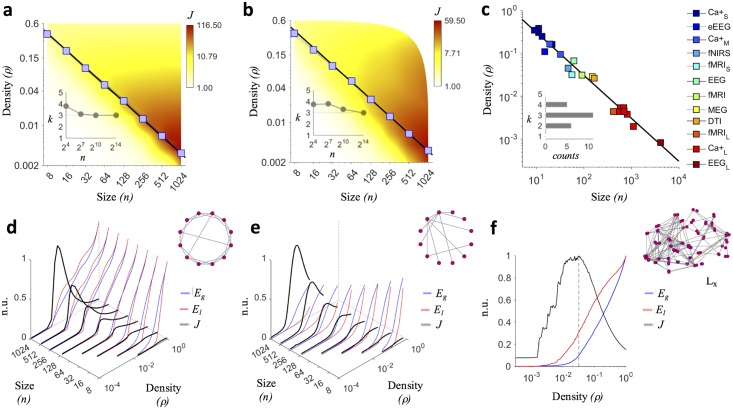
Density threshold in synthetic networks and in brain networks. (**a**–**b**) Blue curves show the trends of the connection density threshold *ρ* for one-hundred generated small-world *p*_*ws*_ = 0.1 and scale-free *m*_*ba*_ = 9 networks along different sizes *n*. Blue squares spot out the average *ρ* values returned by the maximization of *J*. The black line shows the fit *ρ* = *c*/(*n* − 1) to the data, with *c* = 3.258 for small-world networks and *c* = 3.215 for scale-free networks (S1 Table). The background color codes for the average value of the quality function *J*. Insets indicate that the optimal average node degree, corresponding to the density that maximizes *J*, converges to *k* = 3 for large network sizes (*n* = 16834). (**c**) Optimal density values maximizing group-averaged *J* profiles for different brain networks. Imaging connectomes come from previously published studies (Table 1). The fit *ρ* = *c*/(*n* − 1) to the pooled data gives *c* = 3.06 (adjusted *R*^2^ = 0.994). The inset shows a sharp distribution for the corresponding average node degree, with a mode *k* = 3. (**d**–**e**) Average *J* profile (black curves) for simulated small-world and scale-free networks as a function of the network size (*n*) and of the density (*ρ*). *J* values are represented in normalized units (n.u.), having scaled them by the global maximum obtained for *n* = 1024. Blue and red curves show respectively the profiles of global- (*E*_*g*_) and local-efficiency (*E*_*l*_). (**f**) Group-averaged *J* profile for fMRI connectomes (Table 1). The grey dashed line indicates the actual density maximizing *J*, i.e., *ρ* = 0.035, corresponding to an average node degree *k* = 3.115. The graph illustrates the brain network of a representative healthy subject (lateral view, frontal lobe on the left *L*_*x*_).

We confirmed this result (S2a and S2b Fig) through extensive numerical simulations (Materials and Methods), showing that it held true also in more realistic network models, such as in small-world networks [24] (Fig 1a) and in scale-free networks [25] (Fig 1b). For these simulated networks the fitted values varied progressively from *c* = 3.265, in lattices, to *c* = 2.966, in random networks, thus falling within the theoretical range found analytically (S1 Table).

Notably, the optimal density values maximizing *J* emphasized the intrinsic properties (random or regular) of all the implemented synthetic networks in terms of global- and local-efficiency (Fig 1d and 1e and S2d and S2e Fig).

### Density threshold in networks inferred from neuroimaging data

We computed the quality function *J* in both micro- and macro-scale brain networks and we evaluated how the density maximizing *J* scaled as a function of the network size. We considered connectomes used in previously published studies that were obtained with different imaging modalities, from calcium imaging to EEG, and constructed with disparate brain connectivity methods (Table 1).

**Table 1:** Experimental details and network characteristics of imaging connectomes.

Imaging modality	Group(s)	Species	Samples x Group	Condition	Nodes	Connectivity method	Domain	Links	Ref.
Ca^+^_S_	Healthy	Zebrafish	5	Spontaneuous	[9,21]	Granger causality	Time	Directed	[58]
eEEG	Healthy	Rodent	1	Evoked potential	15	Partial directed coherence	Time/Freq. (8 ms/14–29 Hz)	Directed	[59]
Ca^+^_M_	-	Culture	2	Spontaneous	[19,32]	Time delay	Time	Directed	[60]
fNIRS	Healthy	Human	2	Resting state	46	Pearson’s correlation	Time	Undirected	[61]
fMRI_S_	Healthy	Primate	3	Resting state	56	Pearson’s correlation	Time	Undirected	[62]
EEG	Healthy, Stroke	Human	20	Motor imagery	61	Imaginary coherence	Frequency (14–29 Hz)	Undirected	[63]
fMRI	Healthy, Coma	Human	17	Resting state	90	Wavelet correlation	Time	Undirected	[64]
MEG	Healthy, Epilepsy	Human	5	Resting state	149	Spectral coherence	Frequency (5–14 Hz)	Undirected	[65]
DTI	Healthy, Epilepsy	Human	19	-	164	Fractional anisotropy	-	Undirected	[66]
fMRI_L_	Healthy, Coma	Human	17	Resting state	417	Wavelet correlation	Time	Undirected	[64]
Ca^+^_L_	-	Culture	6	Spontaneous	[562,1107]	Time delay	Time	Directed	[60]
EEG_L_	Healthy	Human	5	Motor execution	4094	Imaginary Coherence	Frequency (13–30 Hz)	Undirected	[67]

For each connectome we applied a standard density-based thresholding. We started with the empty network by removing all the links (*ρ* = 0). Then, we reinserted and binarized one link at time, from the strongest to the weakest, until we obtained the maximally dense network (*ρ* = 1). At each step we computed *J* and we recorded its profile as a function of *ρ*. The pooled density values, as returned by the maximization of the healthy group-averaged *J* profile in each modality (see Fig 1f for one representative), followed a power law comparable to the one that we reported for synthetic networks (Fig 1c). In particular, the fit *ρ* = *c*/(*n* − 1) to the data gave *c* = 3.06 with an adjusted r-square *R*^2^ = 0.994. Notably, we obtained a similar scaling (*c* = 2.87 adjusted *R*^2^ = 0.946, S2c Fig) when considering individual *J* profiles (S2f Fig). These results confirm that also for brain networks we can assume that the optimal density threshold maximizing *J* only depends on the network size according to the same rule *ρ* ≃ 3/(*n* − 1).

In conclusion, we introduced a criterion, named efficiency cost optimization (ECO), to select a threshold leading to sparse, yet informative brain networks. Such a threshold is relatively independent of the connectome’s construction and invariant to the underlying network topology so that it can be selected *a-priori* once the number of nodes is known.

### ECO discriminated network properties of different brain states

To illustrate the methodology, we considered connectomes from four different imaging modalities, namely EEG, MEG, fMRI, and DTI (Table 1). Because we do not know the true structure for these connectomes, we evaluated the ability of ECO to discriminate network properties of different brain states, i.e., healthy *versus* diseased, at individual level.

We characterized brain networks by calculating graph quantities at different topological scales, i.e., large (global- and local-efficiency, *E*_*g*_ and *E*_*l*_), intermediate (community partition, *P*; and modularity, *Q*), and small (node degree, *k*_*i*_; and betwenness, *b*_*i*_) (Materials and Methods). To assess network differences between brain states, we measured distances between the values of the graph quantities obtained in the healthy group and those in the diseased group. We adopted the Mirkin index (*MI*) to measure distances between community partitions, and the divergent coefficient (*D*) for other graph quantities (Materials and Methods).

**Fig 2:**
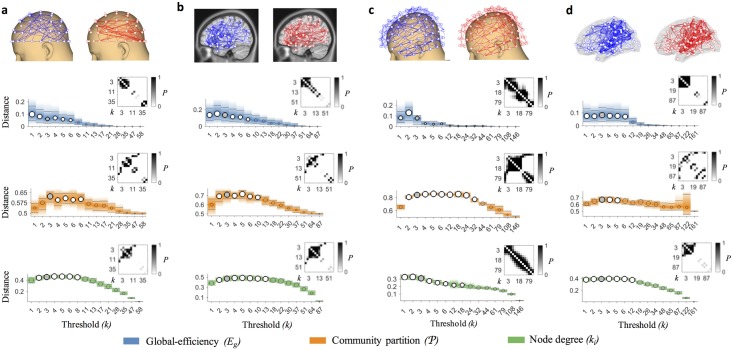
Statistical comparison of brain network distances across thresholds. (**a**–**d**) Top panels show group-averaged connectomes filtered with ECO for the healthy (blue links) and diseased (red links) group, in three representative imaging modalities, i.e., EEG, fMRI, MEG and DTI (Table 1). Lower panels show distances between individual brain network properties across different thresholds for global-efficiency *E*_*g*_, community partition *P*, and node degree vector K = [*k*_1_, …, *k*_*n*_]. Thresholds are given by the average node degree *k*, which corresponds to a connection density *ρ* = *k*/(*n* − 1). Circle sizes are proportional to the median of the graph quantity values; horizontal grey lines correspond to lower and upper quartiles; bar colors shade after quartiles. Overall, the distance significantly depends on the threshold value (Kruskalwallis tests, *P* < 0.01, S2 Table). Grey circles represent distances corresponding to the threshold *k* = 3. White circles denote threshold values for which distances are not significantly different from *k* = 3 (Tukey-Kramer post-hoc tests, *P* ≥ 0.01). Transparent circles denote threshold values for which distances are significantly lower than *k* = 3 (Tukey-Kramer post-hoc test, *P* < 0.01). Insets show the *P*-values resulting from the Tukey-Kramer post-hoc comparison of distances between all the threshold values.

**Fig 3:**
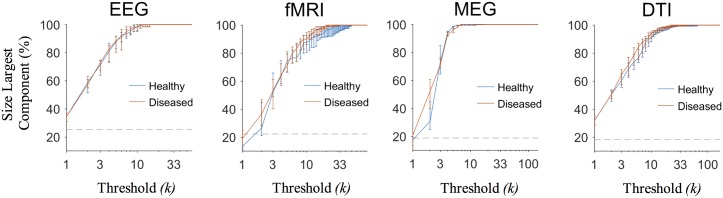
Size of the largest component in brain networks filtered with ECO and statistical comparison between groups. The size of the largest component is given as a percentage of total nodes. Blue lines stand for median values of the healthy group; red lines are median values of the diseased group. Vertical bars denote lower and upper quartiles. The dashed gray line shows the expected size for the giant component in a Erdos-Renyi random graph with *p* = 1/*n*). No statistical between-group differences for any threshold value were reported (Wilcoxon ranks-sum tests, *P* ≥ 0.01).

**Fig 4:**
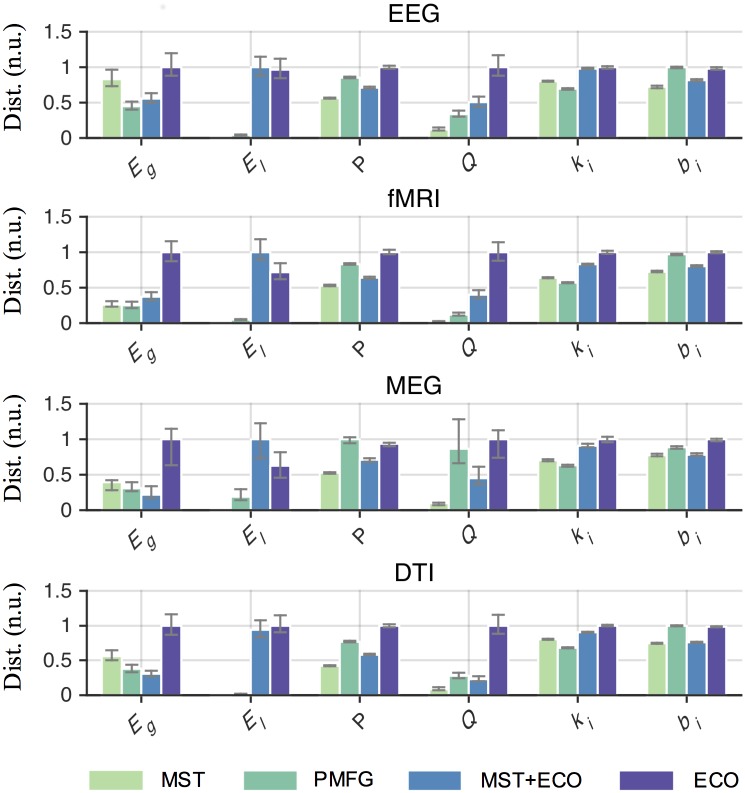
Statistical comparison of brain network distances across filtering methods. Bar plots show the medians of distance between brain network properties of samples in the healthy and diseased group. Vertical bars denote lower and upper quartiles. Medians and quartiles are in normalized units (n.u.) for the sake of representation. Overall, the choice of the filtering method significantly affects distances between samples (Kruskalwallis tests, *P* < 0.01, S3 Table). For all graph quantities, ECO tends to give significantly larger distances as compared to other methods (Tukey-Kramer post hoc tests, *P* < 0.05); in some isolated cases, no significant improvements are reported (S4 Table). By construction, MST gives null distances for local-efficiency as there are no triangles in tree-like networks.

We explored a wide range of density thresholds and, as expected, the value of the threshold affected the ability to separate network properties of different brain states (Kruskalwallis tests *P* < 0.01, S2 Table). Notably, the choice *ρ* = 3/(*n* − 1) resulted among the best candidates in producing larger distances regardless of the graph quantity (Tukey-Kramer post hoc tests *P* < 0.05, Fig 2 and S3 Fig). This outcome was not associated to the possible presence of disconnected components. In all the filtered brain networks the size of the largest component (> 50% of the nodes) did not differ between groups for any threshold value (Wilcoxon rank-sum tests *P* ≥ 0.01, Fig 3). Furthermore, ECO overall outperformed alternative methods, such as the minimum spanning tree (MST) and the planar maximally filtered graph (PMFG) [26], in giving larger distances (Tukey-Kramer post hoc tests *P* < 0.05, Fig 4, S4b Fig, S2 and S3 Tables). Notably, we reported good performance with respect to a hybrid method, named MST+ECO, where we added the remaining strongest links to the backbone obtained with MST, in order to reach the same average node degree as ECO, i.e. *k* = 3 (Tukey-Kramer post hoc tests *P* < 0.05, S3 Table).

**Fig 5:**
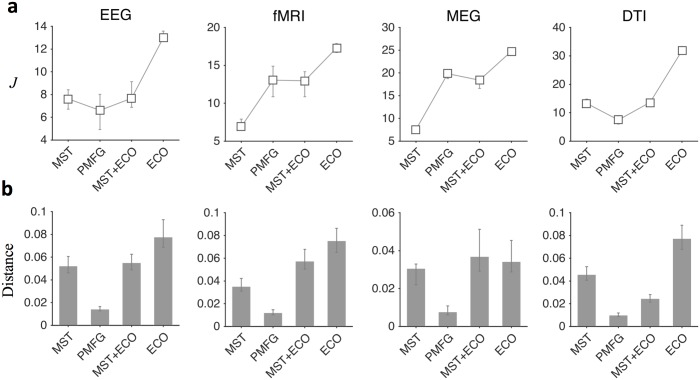
Statistical comparison of *J* values and distances across different thresholding methods. Panel a) White squares show the medians of the *J* values of all the subjects in the two groups. Vertical bars denote the 5*th* and 95*th* percentiles. Panel b) Grey bars show the medians of the distances between samples (individuals) of different brain states. Vertical bars denote lower and upper quartiles. The choice of the filtering method significantly affects the *J* values and the respective distances between samples (Kruskalwallis tests, *P* < 0.01 for both *J* values and related distances, S3 Table). Overall, ECO gives significantly larger values as compared to the other methods (Tukey-Kramer post hoc tests, *P* < 0.05); in some isolated cases no significant improvements are reported (S4 Table).

Finally, brain networks filtered with ECO were more efficient (Fig 5a) and exhibited *J* values that better separated different brain states (Fig 5b) as compared to the other filtering methods (Tukey-Kramer post hoc tests *P* < 0.05, S3 Table).

## Discussion

We introduced ECO to filter information in networks whose links are predictions, and not direct measures, of connectivity between biological components, such as brain regions. Conventional approaches evaluate brain network properties across a large and arbitrary number of thresholds [27]. Eventually, they select a representative threshold *a-posteriori* that maximizes the separation between different brain states [11]. ECO allows to select an objective threshold *a-priori*, thus reducing the computational burden associated with typical iterative approaches.

Other methods, similar in purpose to ECO, impose unnatural constraints on the filtered network. The minimum spanning tree (MST), for instance, leads to brain networks with a null clustering coefficient [28]. The planar maximally filtered graph (PMFG) tries to alleviate this bias by allowing closed loops, but still forces planarity [26]. Conversely, ECO does not impose structural constraints, apart from favoring sparsity, and lets the intrinsic structure to emerge as illustrated in synthetic networks with known topological organization (Fig 1d and 1e, S2d and S2e Fig). This appears an important feature as different brain states (e.g., diseased *versus* healthy) are often characterized by networks with different topological orders (more random or more regular) depending on the underlying physiopathological neural mechanism [6].

Overall, results obtained with ECO improved the separation of all the considered network properties between different brain states as compared to other thresholds or filtering methods. In general, this does not necessarily imply a significant group difference for each graph quantity. Instead, it means that if there are underlying network differences, then ECO would be able to point them out.

Maximizing global- and local-efficiency with respect to connection density can be seen as a way to emphasize the integration and segregation properties of a connectome [29] while keeping a biologically plausible wiring cost. This rationale dovetails with current evidence showing that advantageous topological properties, such as economic small-world architectures [21], tend to be maximized in brain networks, and that, in general, sparsity increases robustness of complex systems [30].

Using ECO, networks will have a total number of links *m* that scales with the number of nodes as *m* = *cn*, with *c* ≃ 3/2. Put differently, the resulting connection density follows a fractal scaling regardless of the network size according to the power-law *ρ* ≃ 3*n*^−1^. Fractal scaling of size and density in self-organized systems has been recently reported and advocated as an important organizational principle to ensure optimal network functioning [31]. Although beyond the scope of this methodological study, we speculate that such characteristic scaling could result, at least for neuronal systems, from a natural optimization of the network efficiency and cost [21].

ECO makes use of density thresholds. Hence, networks having same number of nodes, will have, after pruning, the same number of links. On the one hand, this ensures that differences between network properties are not merely due to differences in the connection density [18]. On the other hand, ECO does not allow a direct evaluation of neural processes altering the number of links; however it does inform on the possible (re)organizational mechanisms.

Finally, it is important to notice that while ECO exhibits several advantageous features, it also has some limitations as described in the following section.

### Methodological considerations

ECO is based on a graph theoretic approach and cannot filter out possible false positives (i.e., spurious links) resulting from biased brain connectivity estimates [7, 11]. Our criterion admits that the weighted links of the raw networks had been previously validated, either maintained or canceled. Some inference methods [32, 33] and group-based approaches [34] naturally produce sparse brain networks. In these cases, ECO would still apply as long as there is enough information to filter, i.e., a number of links $m\geq \frac{3}{2}n$.

By construction, brain networks filtered with ECO (*k* ≃ 3) are less sparse than networks filtered with MST (*k* ≤ 2). However, differently from MST and PMFG, ECO does not guarantee the connectedness of the pruned networks, which can be indeed fragmented (S5 Fig). Whether this condition leads to a more realistic representation of connectomes, especially for large *n*, we cannot say. Current literature tends to focus on thresholded brain networks which are slightly denser than ECO, with 0.05 ≤ *ρ* ≤ 0.3 [35]. However, little is known on how this range depends on the number of brain nodes and future studies will have to ascertain if and how the choice of a specific threshold can give more accurate results. Here, we showed that the size of the largest components contained in average more than the 50% of the nodes (Fig 3). Therefore, caution should be used in the evaluation of the resulting network properties and, whenever possible, using graph quantities that can handle networks with disconnected nodes (e.g., the harmonic mean of the shortest path lengths [36]) appears more appropriate.

Finally, other combinations could have been considered when conceiving the quality function *J*. For example, in [37] authors introduced the cost-efficiency *E*_*g*_ − *ρ*, which, however, did not include the clustering counterpart. This quality function, as well as other ones that we investigated, did not exhibit meaningful analytic solutions and was therefore excluded as a possible alternative (S2 Appendix). A more general formulation would include a scaling factor in the numerator, like for example 2[*αE*_*g*_ + (1 − *α*)*E*_*l*_] where *α* is a control parameter ranging from 0 to 1. We proved analytically that, for both regular lattices and random graphs, the optimal density that maximizes the corresponding quality function remained $\rho ≃\frac{3}{n-1}$ regardless of the *α* value (S3 Appendix). We confirmed this result through numerical simulations also in small-world and scale-free networks (S4 Fig) where the optimal density maximizing *J* corresponded to an average node degree *k* ≃ 3, except when *α* → 1 in lattices and *α* → 0 in random networks.

Taken together, these findings indicate that the density threshold given by ECO is relatively invariant to the specific value we assigned to the parameter *α*. The advantage of considering our quality function is that *i)* it did not depend on external parameters, *ii)* we could derive analytically the optimal *ρ* values for lattices and random networks, and *iii)* the density values obtained by maximizing *J* in real brain networks fitted the power-law that we found analytically and were able to separate different brain states. Despite these advantages, we notice that ECO could not be the definitive solution to the problem of thresholding in imaging connectomics. Other methods, possibly inspired by biology, are likely to be developed in the future and validation benchmarks will be crucial to evaluate their potential.

### Future directions

ECO is founded on asymptotic results in unweighted network models. Its natural application implies binarization after thresholding, a procedure widely adopted to mitigate the uncertainty carried by the weights estimated from neuroimaging data [4, 11]. Further work is needed to clarify how ECO can be extended to weighted networks, where the asymptotic expression of topological properties is less straightforward.

Interactions between biological components are not constant and need to dynamically vary to accomplish internal regulation and external function [38–40]. In neuroscience, functional brain connectivity exhibits rich temporal dynamics that are fundamental for human cognition and complex behavior [41–44]. Further studies should aim to elucidate if and how brain network differences highlighted by ECO change over time.

### Conclusion

We introduced ECO as a possible method for filtering information in imaging connectomes. Concrete applications range from cognitive to clinical and computational neuroscience. Given its generality, we anticipate that ECO can also serve to facilitate the analysis of interconnected systems where the need of sparsity is plausible and the links are weighted estimates of connectivity. This is, for example, the case of functional networks in system biology, where links are typically derived from transcriptional or phenotypic profiling, and genetic interactions [3].

## Materials and Methods

### On the quality function *J*

The expression of *J* can be seen as a particular case of a general family of functions of the form *f*(*E*_*g*_, *E*_*l*_, *ρ*). Here, we defined *J* as a ratio to measure the incidence of the density on the network efficiency both at global and local scale. Indeed, we were interested in a relative measure that could tell the network efficiency changes per unit of density. In addition, we did not weight the global- and local-efficiency in the numerator. While, in general, a scaling factor might be necessary to normalize changes between different graph quantities [45], here both *E*_*g*_ and *E*_*l*_ range between 0 and 1 and are formulated in terms of the same concept, namely the efficiency (at global and local scale) between nodes [22]. We remind to S3 Appendix and S4 Fig for more details on the introduction of a scaling parameter.

By looking at Eq (1), we have that when *ρ* = 0, then both global- *E*_*g*_ and local-efficiency *E*_*l*_ are null leading to an indefinite form. As density slightly increases (0 < *ρ* < *ϵ*, with *ϵ* sufficiently small) it can be demonstrated that *J* tends to 1. Indeed, in this range, the probability to find at least three nodes connected together (a triangle) is extremely low. By definition, *E*_*l*_ = 0 in absence of at least one triangle [22] and therefore *J* ≃ *E*_*g*_/*ρ*. By considering the definitions of *E*_*g*_ and *ρ*, this quantity can be rewritten as $E_{g}/\rho =1/m\sum_{i\neq j}^{n}1/d_{i,j}$, where *m* is the number of existing links and *d*_*i*,*j*_ is the distance between the nodes *i* and *j*. In a generic network with *m* links there are at least *m* pairs of nodes directly connected (i.e., *d*_*i*,*j*_ = 1). This means that the sum in the latter equation is bounded from below by *m* in the case of isolated pairs of connected nodes (*m* = *n*/2) or in the trivial case of *m* = 1. It follows that *J* → 1 when there are relatively few links in a network.

When *ρ* tends to 1, it is trivial to see from Eq (1) that *J* → 2, as both *E*_*g*_ and *E*_*l*_ tend to one. For intermediate density ranges (*ϵ* < *ρ* ≪ 1 − *ϵ*) the analytic estimate of *J* is not trivial since *E*_*g*_ and *E*_*l*_ depend on the network topology which is, in general, unknown.

### Numerical simulations for small-world and scale-free networks

Small-world networks were generated according to the Watts-Strogatz (WS) model [24] with a rewiring probability *p*_*ws*_ = 0.1. Scale-free networks were generated according to the Barabasi-Albert (BA) model [25].

In the first simulation, we considered undirected networks. We varied both the network size and the average node degree, i.e., *n* = 16, 128, 1024, 16384 and *k* = 1, 2, 3, 4, 5. In the WS models, *k* is even accounting for the number of both left and right neighbors of the nodes in the initial lattice. To obtain small-world networks with *k* odd, we first generated lattices with *k* even and then, for each odd node (e.g., 1, 3, …), we removed the link with its left farthest neighbor. This procedure removes in total *n*/2 links leading to a new average node degree *k*′ = *k* − 1, while keeping a regular structure. As for BA models, we set the number of links in the preferential attachment *m*_*ba*_ = 3 and the initial seed was a fully connected network of *n*_0_ = *m*_*ba*_ nodes. This setting generated scale-free networks with *k* = 6 − 12/*n*, that is *k* ≥ 5 regardless of the selected network size. We then removed at random the exceeding number of links until we reached the desired *k* value. This procedure had the advantage to preserve the original scale-free structure.

In the second simulation, we considered directed networks to confirm and extend the results we obtained for undirected WS and BA networks. We selected eight representative network sizes, i.e., *n* = 8, 16, 32, 64, 128, 256, 512, 1024 covering the typical size of most current imaging connectomes, and we varied the connection density. Specifically, we performed a two-step procedure:

1. We fixed one-hundred *ρ* values quadratically spaced within the entire available density interval.
2. After having identified the optimal ρ* maximizing *J*, we performed a refined research among one-hundred new values linearly spaced between the density values, in step 1, before and after ρ*.

For WS models, initial lattices had *k* equal to the nearest even integer equal or higher than *ρ*(*n* − 1), with *ρ* ∈ (0, 1). For BA models, the number of attaching links was *m*_*ba*_ = log_2_ *n* to ensure an initial relatively high density; the seed was a fully connected network of *n*_0_ = *m*_*ba*_ nodes. By construction $\rho \in (0,\frac{2m_{ba}n+m_{0}}{n(n-1)})$, where *m*_0_ = *n*_0_(*n*_0_ − 1)/2 is total number of links in the initial seed. For both models, we then removed at random the exceeding links until we reached the desired density value. For both simulation we generated one-hundred sample networks.

### Graph analysis of brain networks

Complex networks can be analyzed by a plethora of graph quantities characterizing different topological properties [46]. Here, we considered a subset of representative ones which have been shown to be relevant for brain network analysis [47]. To characterize the entire brain network (i.e., large-scale topology), we used global- and local-efficiency, which respectively read:

$$\begin{array}{c} \begin{array}{cc} E_{g} & =\frac{2}{n(n-1)}\sum_{i\neq j}^{n}\frac{1}{d_{ij}}\\ E_{l} & =\frac{1}{n}\sum_{i=1}^{n}E_{g}\left(i\right) \end{array} \end{array}$$ (2)

where *d*_*ij*_ is the length of the shortest path between nodes *i* and *j*, and *E*_*g*_(*i*) is the global-efficiency of the *i*th subgraph of the network [22].

To characterize modules, or clusters, of brain regions with dense connections internally and sparser connections between groups (i.e., mid-scale topology), we evaluated the community structure of the brain network [4]. We extracted the partition *P* of the network into modules by means of the Newman’s spectral algorithm maximizing the modularity:

$$Q=\frac{1}{2m}Tr\left(G^{T}MG\right)$$ (3)

where **G** is the (non-square) matrix having elements *G*_*ig*_ = 1 if node *i* belongs to cluster *g* and zero otherwise, and **M** is the so-called modularity matrix [48].

To characterize individual brain areas (i.e., small-scale topology), we measured the centrality of the nodes in the brain network by means of the node degree and of the node betwenness, which respectively read:

$$\begin{array}{c} \begin{array}{cc} k_{i} & =\sum_{j\neq i}^{n}A_{ij}\\ b_{i} & =\sum_{j\neq i\neq h}\frac{\sigma_{jh}\left(i\right)}{\sigma_{jh}} \end{array} \end{array}$$ (4)

where the element of the adjacency matrix *A*_*ij*_ = 1 if there is a link between node *i* and *j*, zero otherwise; and where *σ*_*jh*_ is the total number of shortest paths between nodes *j* and *h*, while *σ*_*jh*_(*i*) is the number of those paths that pass through *i*.

These quantities represent a small subset of all the possible metrics available in the market. Nevertheless, these are among the most adopted in network neuroscience thanks to their interpretability in terms of connectivity at different topological levels (e.g., network, modules, nodes) [4, 11, 27, 49–51].

### Distances between samples and statistical analysis

To assess brain network differences between individuals (or samples) in the two groups, we measured the distance between the respective values obtained for each graph quantity. We used the Mirkin index to compute distances between two network partitions *P*_*u*_ and *P*_*v*_:

$$MI\left(P_{u},P_{v}\right)=2\left(n_{01}+n_{10}\right)$$ (5)

where *n*_01_ is the number of pairs of nodes in the same cluster under *P*_*v*_ but not under *P*_*u*_; and *n*_10_ is the number of pairs in the same cluster under *P*_*u*_ but not under *P*_*v*_ [52]. The Mirkin index is an adjusted form of the well-known Rand index and it assumes null value for identical clusterings and 1 for totally different clusterings [52]. It corresponds to the Hamming distance between the binary vector representation of each partition. Although this measure can be sensitive to the cluster sizes, it has the advantage of being a metric on the space of the clustering partitions [53].

For all other graph quantities, we used the divergent coefficient [54]:

$$D\left(X_{u},X_{v}\right)=\sqrt{\frac{1}{M}\sum_{m=1}^{M}{\left(\frac{x_{u,m}-x_{v,m}}{x_{u,m}+x_{v,m}}\right)}^{2}}$$ (6)

where X_*u*_ = [*x*_*u*,1_, *x*_*u*,2_, …, *x*_*u*,*M*_] and X_*v*_ = [*x*_*v*,1_, *x*_*v*,2_, …, *x*_*v*,*M*_], contain the value(s) of the graph quantity for the *u*th and *v*th sample. Notably, *M* = 1 for global-, local-efficiency and modularity (i.e., *E*_*g*_, *E*_*l*_, *Q*). *M* = *n* for the node degree vector K = [*k*_1_, *k*_2_, …, *k*_*n*_] and the node betweenness vector B = [*b*_1_, *b*_2_, …, *b*_*n*_]. The divergent coefficient is a L2-norm distance similar to Euclidean distance but with a normalizing factor which is used for multidimensional scaling [55]. It ranges between 0 (equal multidimensional distribution of the features) and 1 (totally heterogeneous multidimensional distribution). This coefficient is a metric in the Euclidean space when all the values of the features are positive, as for our graph quantities [56]. Both Mirkin index and divergent coefficient are therefore metrics normalized between 0 and 1, allowing for a coherent analysis across different imaging modalities and threshold values.

We used Kruskal–Wallis one-way analysis of variance, with a 0.01 statistical threshold, to evaluate the overall effect of different thresholds, or filtering methods (i.e., MST, PMFG) on distances between individuals. A Tukey-Kramer multiple comparison post hoc test was then used to determine specific differences between pairs of thresholds or methods [57]. Here the statistical threshold was fixed to 0.05.

## Supporting Information

S1 Appendix (PDF)

S2 Appendix (PDF)

S3 Appendix (PDF)

S1 Fig From raw imaging connectomes to binary brain networks. As a result of measurements, a raw imaging connectome is mathematically described by a full and weighted connectivity matrix **W**. To obtain a sparse brain network, the raw information is filtered and binarized by applying a threshold either on the weights (i.e., the connectivity strength) or on the percentage (i.e., the connection density) of strongest weights to retain in the adjacency matrix **A**. Data showed here are just for illustrative purposes and not used in the rest of the paper.
(TIFF)

S2 Fig Density threshold in synthetic networks and in brain networks. (**a**–**b**) Blue curves show the trends of the optimal density *ρ* that maximizes *J* for one-hundred generated lattices and random networks along different sizes *n*. Blue squares spot out the corresponding average values. The black line shows the fit *ρ* = *c*/(*n* − 1) to the data, with *c* = 3.265 for lattices and *c* = 2.966 for random networks (S1 Table). The background color codes for the average value of the quality function *J*. Insets indicate that the average node degree corresponding to the optimal *ρ* maximizing *J* converges to *k* = 3 for large network sizes (*n* = 16834). (**c**) Optimal density maximizing individual *J* profiles for different brain networks. Imaging connectomes come from previously published studies (Table 1). A larger variability can be observed with respect to the values we obtained when considering group-averaged *J* profiles Fig 1c. The inset confirms a more uniform distribution for the average node degree corresponding the optimal *ρ* that maximizes *J*. Nevertheless, the fit *ρ* = *c*/(*n* − 1) to the pooled data gives *c* = 2.87 (adjusted *R*^2^ = 0.946), which is in practice very close to *k* = 3. (**d**–**e**) Average *J* profile (black curves) for simulated lattices and random networks as a function of the network size (*n*) and of the density (*ρ*). *J* values are represented in normalized units (n.u.), having scaled them by the global maximum obtained for *n* = 1024. Blue and red curves show respectively the profiles of the global- (*E*_*g*_) and local-efficiency (*E*_*l*_). (**f**) Individual *J* profile for a representative fMRI connectome (Table 1). The grey dashed line indicates the actual density maximizing *J*, i.e., *ρ* = 0.008, corresponding to an average node degree *k* = 0.712. This value was very far from the expected *k* = 3. Indeed, we noticed that for very low density values the intrinsic brain network structure could not completely emerge and spurious peaks could appear. The graph illustrates the brain network of a representative healthy subject (lateral view, frontal lobe on the left *L*_*x*_).
(TIFF)

S3 Fig Statistical comparison of brain network distances across thresholds. Results for for local-efficiency *E*_*l*_, modularity *Q*, and node betwenness vector B = [*b*_1_, …, *b*_*n*_]. Panel (**a**) show distances for EEG connectomes, (**b**)-fMRI, (**c**)-DTI, (**d**)-MEG. Thresholds are given by the average node degree *k*, which corresponds to a connection density *ρ* = *k*/(*n* − 1). Circle sizes are proportional to the median of the graph quantity values; horizontal grey lines correspond to lower and upper quartiles; bar colors shade after quartiles. Overall, the distance significantly depends on the threshold value (Kruskalwallis tests, *P* < 0.01; S2 Table). Grey circles represent distances for the threshold corresponding to *k* = 3. White circles denote threshold values for which distances are not significantly different from *k* = 3 (Tukey-Kramer post-hoc tests, *P* ≥ 0.01). Transparent circles denote threshold values for which distances are significantly lower than *k* = 3 (Tukey-Kramer post-hoc tests, *P* < 0.01). Insets show the *P*-values resulting from the Tukey-Kramer post-hoc comparison of distances between all the threshold values.
(TIFF)

S4 FigOptimal density maximizing parametric quality function in synthetic networks.Background colors code for the average values of $J=2\frac{\alpha E_{g}+\left(1-\alpha \right)E_{l}}{\rho }$ in a logarithmic scale. Synthetic networks, as generated by the models described in the Materials and Methods, have *n* = 512 nodes. White circles spot out the maximum as a function of the parameter *α* (y-axis). The black line shows the density value *ρ* = 3/(*n* − 1) corresponding to *k* = 3 (x-axis).(TIFF)

S5 Fig Density required for connectedness of synthetic networks. Dark grey area corresponds to density values for which random networks do not exhibit a giant component (*ρ* < 1/*n*). Light grey area corresponds to density values for which random networks do exhibit a giant component (*ρ* > 1/*n*). White area corresponds to density values for which random networks are connected (*ρ* > *log*(*n*)/*n*). Colored symbols show the mean connection density values for which the simulated synthetic networks become connected. Black solid line illustrates the connection density *ρ* = 3/*n*. Results show that for large *n*, density values returned by ECO (i.e., ≃ 3/*n*) guarantee the connectedness of the filtered network only if the underlying structure is regular. Indeed, the minimum requirement for connectedness in lattices is *ρ* > 2/*n*.
(TIFF)

S1 Table Statistics of data fitting *ρ* = *c*/(*n* − 1) to synthetic networks. The fit’s constant *c* and the adjusted *R*^2^ coefficient are reported along different network models.
(PDF)

S2 Table *P*-values from Kruskalwallis tests on brain network distances across thresholds. (DOC)

S3 Table *P*-values from Kruskalwallis tests on brain network distances across filtering methods. *J*_*val*_ stand for the actual values (not distances) of the quality function *J*.
(DOC)

S4 Table *P*-values from Tukey-Kramer post-hoc tests on brain network distances across filtering methods. Cross symbols denote no significant differences (*P* > 0.05). For local-efficiency (*E*_*l*_), tests were not performed when comparing ECO *vs* MST, as by construction, MST gives null *E*_*l*_ values. *J*_*val*_ stand for the actual values (not distances) of the quality function *J*.
(DOC)
