# Supplementary material for: A Topological Criterion for Filtering Information in Complex Brain Networks
Source: PLoS Comput Biol. 2017 Jan 11;13(1):e1005305. doi: 10.1371/journal.pcbi.1005305 (PMC5268647; doi:10.1371/journal.pcbi.1005305)
Supplement: S1 Appendix — (PDF) [file pcbi.1005305.s001.pdf]

S1 Appendix

Optimal density maximizing J for regular lattices and random networks.

Global- E_g and local-efficiency E_l can be well approximated, respectively, by the inverse of the characteristic path length L and by the clustering coefficient C [1]. We can then rewrite the quality function (Eq. 1 in the main text) as $J = (L^{-1} + C)/\rho$. In some simple cases, L and C can be expressed as a function of the average node degree k and of the number of nodes n of a network.

In particular, for large 1D regular lattices, corresponding to Watts-Strogatz (WS) model with a rewiring probability $p_{ws} = 0$, we have $L_0 = \frac{n}{2k}$ and $C_0 = \frac{3(k-2)}{4(k-1)}$; while for large random networks ($p_{ws} = 1$), we have $L_1 = \frac{\log n}{\log k}$ and $C_1 = \frac{k}{n}$ [2]. Since the relation $\rho = \frac{k}{n-1}$ holds for any network, we can rewrite the quality function J for regular lattices as:

$$J_0 = 2\frac{(n-1)}{n} + \frac{3(n-1)\rho - 6}{4(n-1)\rho^2 - 4\rho} \tag{S1}$$

and for random networks:

$$J_1 = \frac{\log\left[(n-1)\rho\right]}{\rho \log n} + \frac{(n-1)}{n} \tag{S2}$$

Notice that the leading contribution to J is coming from C in the case of a lattice and from L^{-1} in the case of a random network.

For these synthetic networks we can determine analytically the optimal value of the connection density ρ that maximizes the quality function J. In particular, by deriving with respect to ρ and equating to zero, we obtained for lattices the following solution which satisfies the requirement of connectedness ($\rho \geq 2/n$):

$$\rho_0 = \frac{2+\sqrt{2}}{n-1} \tag{S3}$$

As for random networks we obtained:

$$\rho_1 = \frac{e}{n-1} \tag{S4}$$

It follows from Eq. (S3) and Eq. (S4) that in both cases the optimal density maximizing J can be written as $c/(n-1)$, where the constant c, corresponding to the average node degree k, is approximately equal to 3. Without loss of generality we referred here to undirected networks. For directed networks the same results hold, k corresponding to the average node indegree k_{in} or outdegree k_{out}.

Indeed, in a directed graph the density is $\rho = \frac{m}{n(n-1)}$ and $m = \frac{k_{tot}n}{2}$, where $k_{tot} = k_{in} + k_{out}$. Since in every directed graph $k_{in} = k_{out}$ we can write, for example, $k_{tot} = 2k_{in}$. Hence the density reads as $\rho = \frac{k_{tot}}{2(n-1)} = \frac{k_{in}}{(n-1)}$ which is the same relation we found for undirected graphs but with k_{in} instead of k.

References

1. Latora V, Marchiori M. Efficient behavior of small-world networks. Physical Review Letters. 2001;87(19):198701/1–198701/4.

2. Watts DJ, Strogatz SH. Collective dynamics of 'small-world' networks. Nature. 1998;393(6684):440–442.
