# Supplementary material for: A Topological Criterion for Filtering Information in Complex Brain Networks
Source: PLoS Comput Biol. 2017 Jan 11;13(1):e1005305. doi: 10.1371/journal.pcbi.1005305 (PMC5268647; doi:10.1371/journal.pcbi.1005305)
Supplement: S2 Appendix — (PDF) [file pcbi.1005305.s002.pdf]

S2 Appendix

Optimal density maximizing alternative quality functions.

We considered alternative quality functions combining E_g, E_l and ρ. For each new J, we checked analytically the existence of an optimal density maximizing J in both regular lattices and random networks. We excluded the quality functions for which we obtained indefinite, non-closed or trivial solutions.

- $J = E_g + E_l - \rho \simeq 1/L + C - \rho$

  By substituting the expression of L and C for random networks we obtain:

  $$J_1 = \frac{\log k}{\log n} + \frac{k}{n} - \frac{k}{n-1} \tag{S5}$$

  For the sake of simplicity we solved with respect to k, knowing that $\rho = k/(n-1)$. When derivating and equating to zero we had $k = \frac{n(n-1)}{\log n}$, which leads to the impossible condition $\rho = n/\log n > 1$.

- $J = E_g - \rho \simeq 1/L - \rho$

  By using the expression of L for lattices we obtain:

  $$J_0 = \rho \left(2\frac{(n-1)}{n} - 1 \right) \tag{S6}$$

  When derivating and equating to zero we had $n = 2$ and we cannot solve with respect to ρ.

- $J = E_l - \rho \simeq C - \rho$

  By substituting the expression of C for random networks we obtain:

  $$J_1 = \frac{k}{n} - \frac{k}{n-1} \tag{S7}$$

  When derivating and equating to zero we had $1/n = 1/(n-1)$ and we could not solve with respect to ρ.

- $J = E_g E_l/\rho \simeq (C/L)/\rho$

  By substituting the expression of L and C for random networks we obtain:

  $$J_1 = \frac{n-1}{n \log n} \log k \tag{S8}$$

  When derivating and equating to zero we had the trivial solution $k = 0$.

Similarly, it is easy to prove that neither $J = E_g/\rho$ nor $J = E_l/\rho$ admitted meaningful solutions in lattices or random networks.
