# Supplementary material for: A Topological Criterion for Filtering Information in Complex Brain Networks
Source: PLoS Comput Biol. 2017 Jan 11;13(1):e1005305. doi: 10.1371/journal.pcbi.1005305 (PMC5268647; doi:10.1371/journal.pcbi.1005305)
Supplement: S3 Appendix — (PDF) [file pcbi.1005305.s003.pdf]

S3 Appendix

Optimal density maximizing parametric quality functions.

We considered the following formulation of J by weighting the global- and local-efficiency:

$$J' = 2\frac{\alpha E_g + (1-\alpha)E_l}{\rho} \simeq 2\frac{\alpha/L + (1-\alpha)C}{\rho} \tag{S9}$$

where α is a control parameter ranging between 0 and 1. Notice that when $\alpha = 0.5$ we obtained the original expression of J - Eq. (1) in the main text. By substituting the expression of L and C in regular lattices and in random networks, we basically obtain the same formulas as in Eq. (S1) and (S2) in the S1 Appendix, with the only difference that the two addenda are respectively multiplied by the constant 2α and $2(1-\alpha)$. In both cases, it is trivial to show that when derivating with respect to ρ, and equating to zero, the role of α vanishes.
