# Supplementary figures and images for: A Topological Criterion for Filtering Information in Complex Brain Networks
Source: PLoS Comput Biol. 2017 Jan 11;13(1):e1005305. doi: 10.1371/journal.pcbi.1005305 (PMC5268647; doi:10.1371/journal.pcbi.1005305)

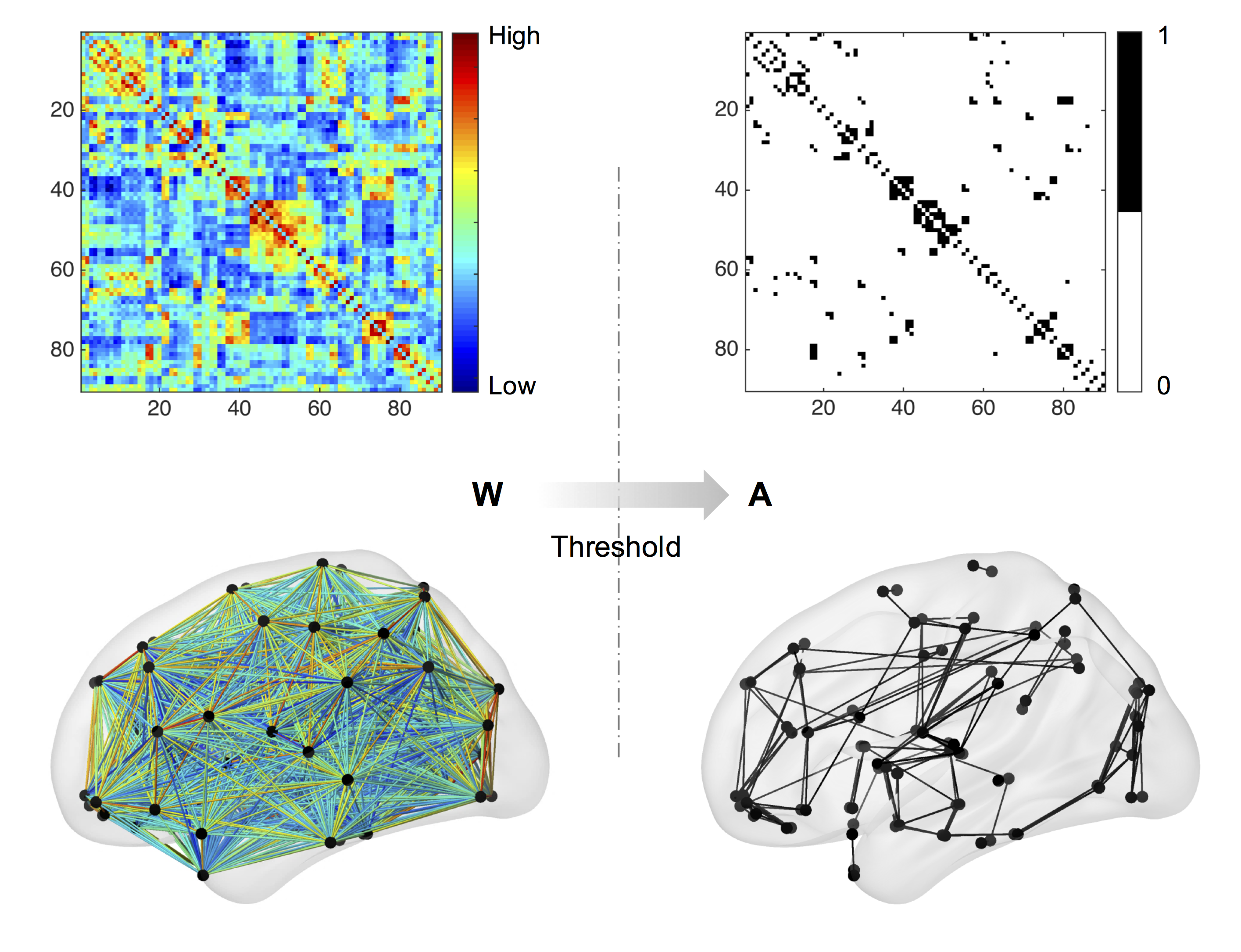

Supplement: S1 Fig — As a result of measurements, a raw imaging connectome is mathematically described by a full and weighted connectivity matrix W. To obtain a sparse brain network, the raw information is filtered and binarized by applying a threshold either on the weights (i.e., the connectivity strength) or on the percentage (i.e., the connection density) of strongest weights to retain in the adjacency matrix A. Data showed here are just for illustrative purposes and not used in the rest of the paper. (TIFF) [file pcbi.1005305.s004.tiff]

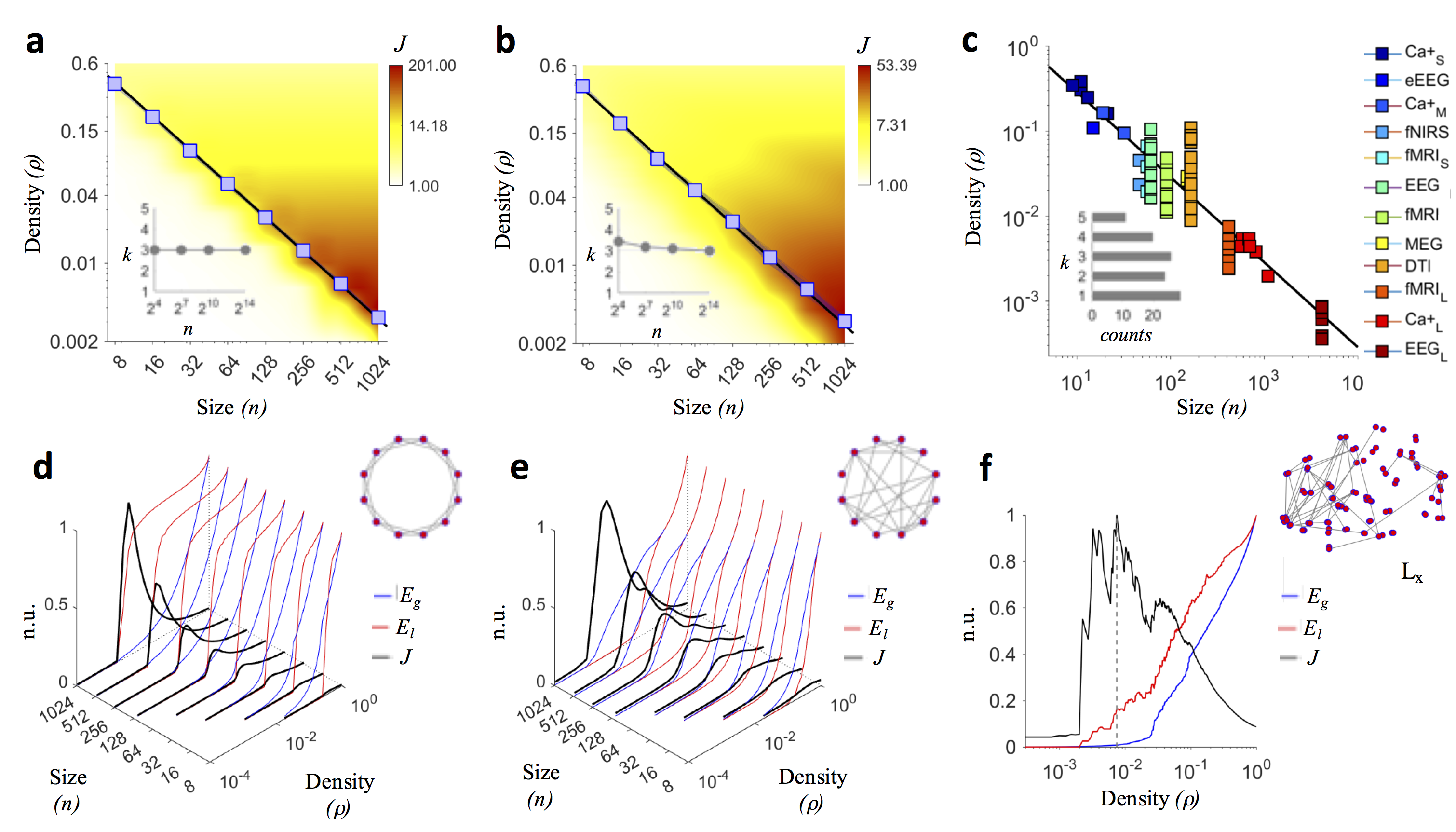

Supplement: S2 Fig — (a–b) Blue curves show the trends of the optimal density ρ that maximizes J for one-hundred generated lattices and random networks along different sizes n. Blue squares spot out the corresponding average values. The black line shows the fit ρ = c/(n − 1) to the data, with c = 3.265 for lattices and c = 2.966 for random networks (S1 Table). The background color codes for the average value of the quality function J. Insets indicate that the average node degree corresponding to the optimal ρ maximizing J converges to k = 3 for large network sizes (n = 16834). (c) Optimal density maximizing individual J profiles for different brain networks. Imaging connectomes come from previously published studies (Table 1). A larger variability can be observed with respect to the values we obtained when considering group-averaged J profiles Fig 1c. The inset confirms a more uniform distribution for the average node degree corresponding the optimal ρ that maximizes J. Nevertheless, the fit ρ = c/(n − 1) to the pooled data gives c = 2.87 (adjusted R2 = 0.946), which is in practice very close to k = 3. (d–e) Average J profile (black curves) for simulated lattices and random networks as a function of the network size (n) and of the density (ρ). J values are represented in normalized units (n.u.), having scaled them by the global maximum obtained for n = 1024. Blue and red curves show respectively the profiles of the global- (Eg) and local-efficiency (El). (f) Individual J profile for a representative fMRI connectome (Table 1). The grey dashed line indicates the actual density maximizing J, i.e., ρ = 0.008, corresponding to an average node degree k = 0.712. This value was very far from the expected k = 3. Indeed, we noticed that for very low density values the intrinsic brain network structure could not completely emerge and spurious peaks could appear. The graph illustrates the brain network of a representative healthy subject (lateral view, frontal lobe on the left Lx). (TIFF) [file pcbi.1005305.s005.tiff]

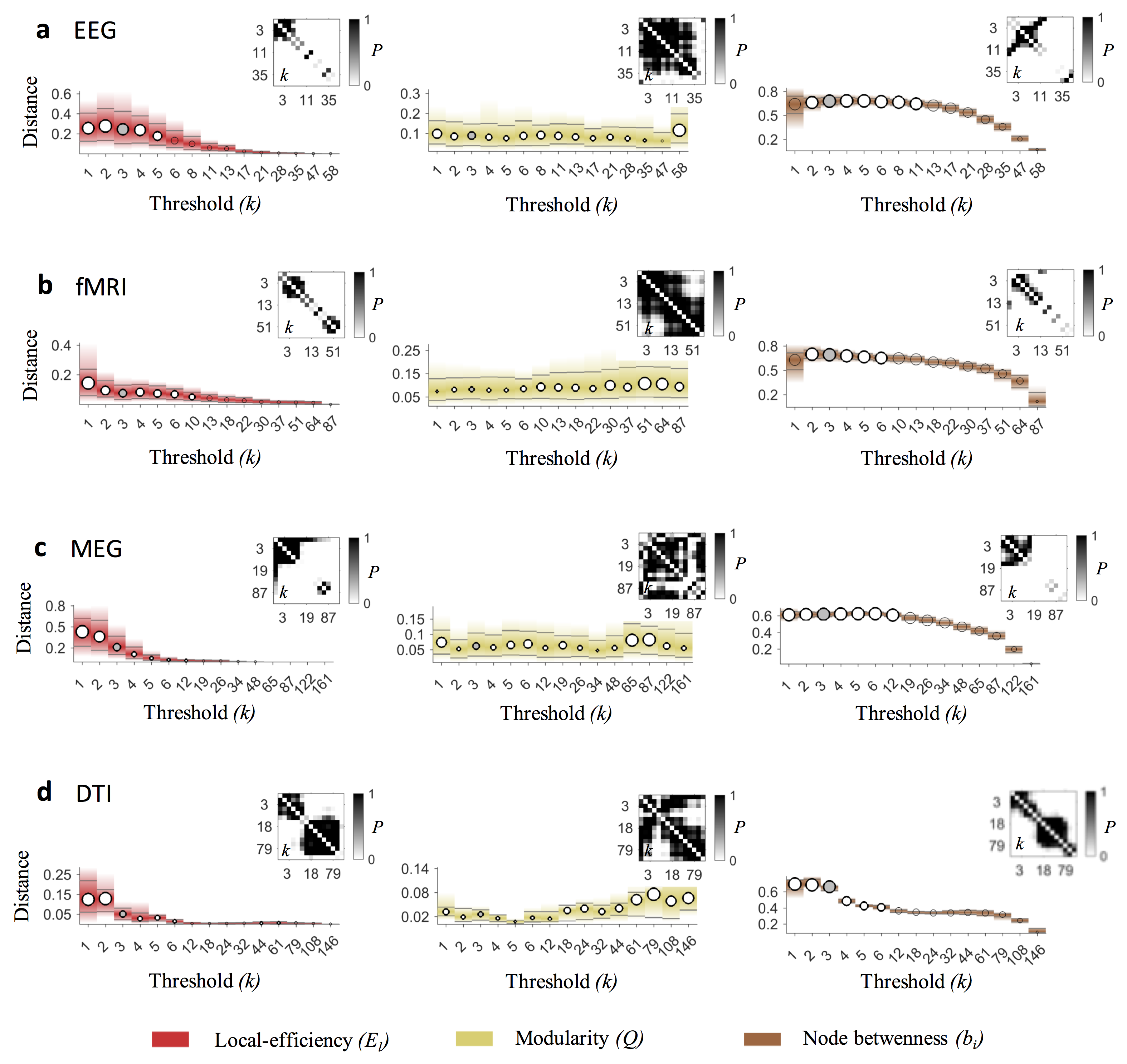

Supplement: S3 Fig — Results for for local-efficiency El, modularity Q, and node betwenness vector B = [b1, …, bn]. Panel (a) show distances for EEG connectomes, (b)-fMRI, (c)-DTI, (d)-MEG. Thresholds are given by the average node degree k, which corresponds to a connection density ρ = k/(n − 1). Circle sizes are proportional to the median of the graph quantity values; horizontal grey lines correspond to lower and upper quartiles; bar colors shade after quartiles. Overall, the distance significantly depends on the threshold value (Kruskalwallis tests, P < 0.01; S2 Table). Grey circles represent distances for the threshold corresponding to k = 3. White circles denote threshold values for which distances are not significantly different from k = 3 (Tukey-Kramer post-hoc tests, P ≥ 0.01). Transparent circles denote threshold values for which distances are significantly lower than k = 3 (Tukey-Kramer post-hoc tests, P < 0.01). Insets show the P-values resulting from the Tukey-Kramer post-hoc comparison of distances between all the threshold values. (TIFF) [file pcbi.1005305.s006.tiff]

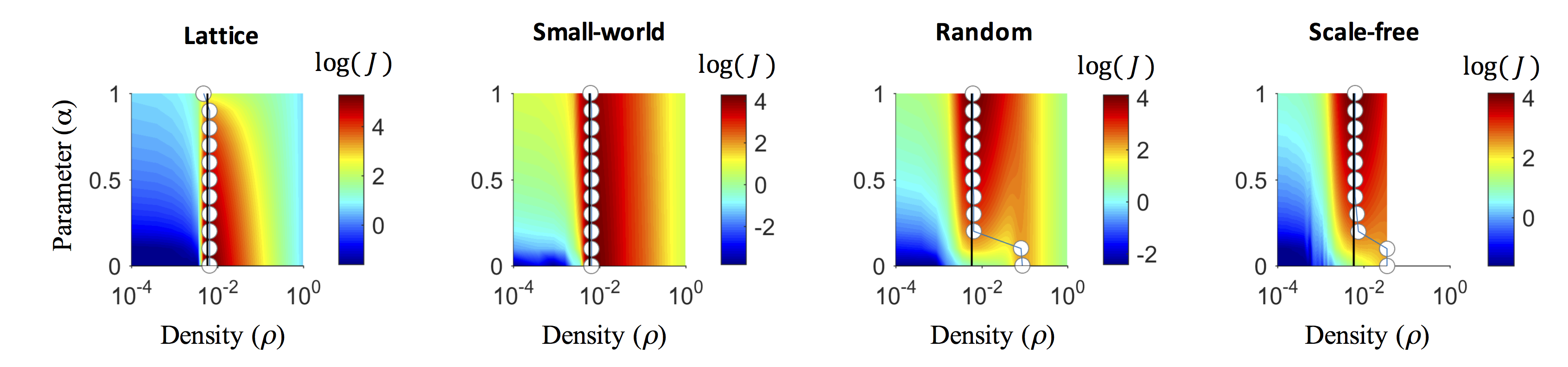

Supplement: S4 Fig — Background colors code for the average values of J=2αEg+(1-α)Elρ in a logarithmic scale. Synthetic networks, as generated by the models described in the Materials and Methods, have n = 512 nodes. White circles spot out the maximum as a function of the parameter α (y-axis). The black line shows the density value ρ = 3/(n − 1) corresponding to k = 3 (x-axis). (TIFF) [file pcbi.1005305.s007.tiff]

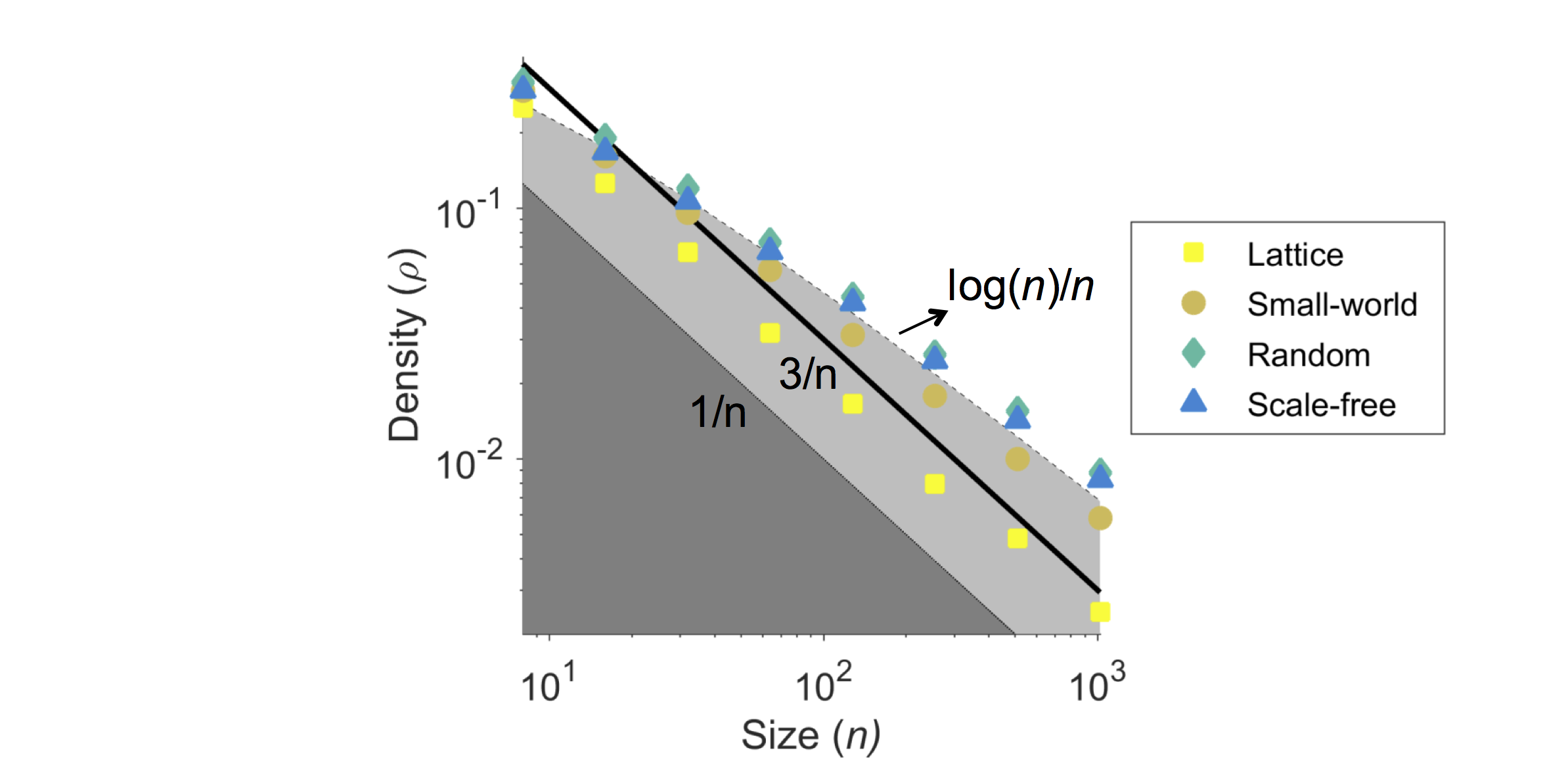

Supplement: S5 Fig — Dark grey area corresponds to density values for which random networks do not exhibit a giant component (ρ < 1/n). Light grey area corresponds to density values for which random networks do exhibit a giant component (ρ > 1/n). White area corresponds to density values for which random networks are connected (ρ > log(n)/n). Colored symbols show the mean connection density values for which the simulated synthetic networks become connected. Black solid line illustrates the connection density ρ = 3/n. Results show that for large n, density values returned by ECO (i.e., ≃ 3/n) guarantee the connectedness of the filtered network only if the underlying structure is regular. Indeed, the minimum requirement for connectedness in lattices is ρ > 2/n. (TIFF) [file pcbi.1005305.s008.tiff]
