# Supplementary material for: A Topological Criterion for Filtering Information in Complex Brain Networks
Source: PLoS Comput Biol. 2017 Jan 11;13(1):e1005305. doi: 10.1371/journal.pcbi.1005305 (PMC5268647; doi:10.1371/journal.pcbi.1005305)
Supplement: S1 Table — The fit’s constant c and the adjusted R2 coefficient are reported along different network models. (PDF) [file pcbi.1005305.s009.pdf]

Table S 1. Statistics of data fitting $\rho = c/(n-1)$ to synthetic networks.

	**Lattice**	**Small-world**	**Random**	**Scale-free**
c	3.265	3.258	2.966	3.215
R^2	0.999	0.997	0.993	0.998

The fit's constant c and the adjusted R^2 coefficient are reported along different network models.
