# Supplementary material for: A Topological Criterion for Filtering Information in Complex Brain Networks
Source: PLoS Comput Biol. 2017 Jan 11;13(1):e1005305. doi: 10.1371/journal.pcbi.1005305 (PMC5268647; doi:10.1371/journal.pcbi.1005305)
Supplement: S2 Table — (DOC) [file pcbi.1005305.s010.doc]

|  | **EEG** | **fMRI** | **MEG** | **DTI** |
| --- | --- | --- | --- | --- |
| *E_g_* | 2.98E-36 | 3.74E-57 | 2.82E-2 | 9.53E-41 |
| *E_l_* | 1.66E-113 | 6.60E-83 | 3.99E-4 | 4.65E-82 |
| P | 1.09E-185 | 5.03E-139 | 8.10E-12 | 1.09E-192 |
| *Q* | 3.51E-106 | 1.94E-130 | 1.15E-10 | 1.07E-62 |
| *k_i_* | 9.57E-214 | 6.70E-151 | 8.25E-14 | 1.21E-203 |
| *b_i_* | 7.19E-162 | 3.49E-125 | 3.38E-11 | 6.35E-184 |
