# Supplementary material for: A Topological Criterion for Filtering Information in Complex Brain Networks
Source: PLoS Comput Biol. 2017 Jan 11;13(1):e1005305. doi: 10.1371/journal.pcbi.1005305 (PMC5268647; doi:10.1371/journal.pcbi.1005305)
Supplement: S3 Table — Jval stand for the actual values (not distances) of the quality function J. (DOC) [file pcbi.1005305.s011.doc]

|  | **EEG** | **fMRI** | **MEG** | **DTI** |
| --- | --- | --- | --- | --- |
| *E_g_* | 5.24E-41 | 8.41E-61 | 9.51E-3 | 6.92E-65 |
| *E_l_* | 4.82E-152 | 1.90E-115 | 9.65E-7 | 3.11E-99 |
| P | 2.15E-259 | 5.04E-188 | 3.71E-17 | 1.27E-267 |
| *Q* | 1.95E-118 | 4.88E-164 | 3.11E-11 | 5.30E-76 |
| *k_i_* | 1.41E-276 | 9.61E-216 | 8.52E-18 | 1.94E-268 |
| *b_i_* | 3.90E-225 | 1.97E-186 | 1.43E-13 | 2.30E-245 |
| *J* | 6.50E-103 | 2.30E-83 | 1.70E-6 | 2.31E-119 |
| *J_val_* | 8.99E-13 | 3.16E-12 | 4.71E-04 | 1.13E-13 |
