# Supplementary material for: A Topological Criterion for Filtering Information in Complex Brain Networks
Source: PLoS Comput Biol. 2017 Jan 11;13(1):e1005305. doi: 10.1371/journal.pcbi.1005305 (PMC5268647; doi:10.1371/journal.pcbi.1005305)
Supplement: S4 Table — Cross symbols denote no significant differences (P > 0.05). For local-efficiency (El), tests were not performed when comparing ECO vs MST, as by construction, MST gives null El values. Jval stand for the actual values (not distances) of the quality function J. (DOC) [file pcbi.1005305.s012.doc]

|  | EEG | | | fMRI | | | MEG | | | DTI | | |
| --- | --- | --- | --- | --- | --- | --- | --- | --- | --- | --- | --- | --- |
|  | **MST** | **PMFG** | **MST+ECO** | **MST** | **PMFG** | **MST+ECO** | **MST** | **PMFG** | **MST+ECO** | **MST** | **PMFG** | **MST+ECO** |
| *E_g_* | 0.83E-03 | 0.37E-9 | 0.37E-9 | 0.37E-8 | 0.37E-8 | 0.37E-8 | 0.0671 ^x^ | 0.2072 ^x^ | 0.0110 | 0.37E-8 | 0.37E-8 | 0.37E-8 |
| *E_l_* | - | 0.37E-9 | 0.8152 ^x^ | - | 0.37E-9 | 0.51E-3 | - | 0.0031 | 0.1330 ^x^ | - | 0.37E-09 | 0.915 ^x^ |
| P | 0.37E-8 | 0.37E-8 | 0.37E-8 | 0.37E-8 | 0.47E-8 | 0.37E-8 | 0.37E-9 | 0.7486 ^x^ | 1.00E-4 | 0.37E-8 | 0.37E-8 | 0.37E-8 |
| *Q* | 0.37E-8 | 0.37E-8 | 0.37E-8 | 0.37E-8 | 0.37E-8 | 0.37E-8 | 0.37E-9 | 0.8815 ^x^ | 0.2562 ^x^ | 0.37E-8 | 0.37E-8 | 0.37E-8 |
| *k_i_* | 0.37E-9 | 0.37E-9 | 0.9451 ^x^ | 0.37E-8 | 0.37E-8 | 0.37E-8 | 0.37E-9 | 0.37E-9 | 0.4511 ^x^ | 0.37E-8 | 0.37E-8 | 0.37E-8 |
| *b_i_* | 0.37E-9 | 0.1713 ^x^ | 0.37E-9 | 0.37E-9 | 0.2442 ^x^ | 0.37E-9 | 0.37E-9 | 0.0239 | 0.37E-9 | 0.37E-9 | 0.1861^x^ | 0.37E-9 |
| *J* | 0.09E-6 | 0.04E-6 | 0.27E-5 | 0.37E-9 | 0.37E-9 | 0.1276 ^x^ | 0.2929 ^x^ | 0.37E-9 | 0.9952 ^x^ | 0.24E-6 | 0.04E-7 | 0.04E-7 |
| *J_val_* | 0.01E-03 | 0.01E-4 | 0.43E-3 | 0.37E-9 | 0.0018 | 0.0005 | 0.0004 | 0.5387 ^x^ | 0.0378 | 0.24E-3 | 0.01E-4 | 0.66E-3 |
